# Histomorphological differentiation of the skin of leopard (*Panthera pardus*), leopard cat (*Prionailurus bengalensis*), Bengal tiger (*Panthera tigris)*, and golden jackal (*Canis aureus*)

**DOI:** 10.14202/vetworld.2020.827-832

**Published:** 2020-04-30

**Authors:** Chukkath Vijayan Rajani, Harshad Sudhir Patki, Patgiri Simanta, Kalaripparambath Surjith, Padinjare Melepat Deepa, Mampillikalam Pradeep

**Affiliations:** 1 Department of Veterinary Anatomy and Histology, College of Veterinary and Animal Sciences, Wayanad, Kerala, India; 2 Department of Veterinary Epidemiology and Preventive Medicine, College of Veterinary and Animal Sciences, Wayanad, Kerala, India; 3 Department of Veterinary Pathology, College of Veterinary and Animal Sciences, Wayanad, Kerala, India

**Keywords:** Bengal tiger, golden jackal, leopard cat, leopard, skin

## Abstract

**Background and Aim:**

Leopard (*Panthera pardus*), leopard cat (*Prionailurus bengalensis*) Bengal tiger (*Panthera tigris*), and golden jackal (*Canis aureus*) are carnivores. Leopard and Bengal tiger are listed in the red list as vulnerable species by the International Union for Conservation of Nature and Natural resources. Leopard cat and golden jackal are grouped under animals of least concern. A wide variation exists in the structure of the skin and pattern of hair follicles among domestic and wild mammals. Thus, the study aims to create a baseline data on the skin of leopard, leopard cat, Bengal tiger, and golden jackal and the data so obtained may form an indispensable tool in wildlife forensics.

**Materials and Methods:**

Skin samples of leopard (n=3), leopard cat (n=4), Bengal tiger (n=3), and golden jackal (n=4) were collected from the Department of Pathology, College of Veterinary and Animal Sciences, Pookode. The samples were processed for paraffin embedding. Horizontal and vertical sections of 5 µm thickness were used for histological staining techniques. Observations on the layers and features of epidermis, hair follicle pattern and glands, namely, sweat and sebaceous were recorded.

**Results:**

Skin comprised an outer epidermis and an inner dermis. Keratinized stratified squamous epithelium made up the epidermis. Stratum basale, stratum spinosum, stratum granulosum, and stratum corneum were discernible in leopard, Bengal tiger, and golden jackal. In leopard cat, stratum basale, stratum spinosum, and stratum corneum were present. Compound hair follicles were a characteristic feature of all species. However, the pattern varied. In leopard, leopard cat and Bengal tiger, a single large primary guard hair was encircled by compound follicles. The number of surrounding compound follicles ranged between five to seven in leopard, two to five in leopard cat, and three to seven in Bengal tiger. Each compound follicle, in turn contained, one to two coarse primary hair follicles and several fine secondary hair follicles. Compound follicles arranged as clusters of three were a salient attribute in jackal. The central follicle was comparatively larger than the lateral ones. Each compound follicle comprised a single long, primary hair, and six to eight smaller secondary hairs.

**Conclusion:**

Histological variation in the skin of the leopard, leopard cat, Bengal tiger, and golden jackal was established. The data form a valuable basis for comparative histology of wild carnivores. Further, the data may be of value in the identification of the unknown skin samples of wild carnivores.

## Introduction

Leopard (*Panthera pardus*), leopard cat (*Prionailurus bengalensis*), Bengal tiger (*Panthera tigris*), and golden jackal (*Canis aureus*) are grouped under the order Carnivora. Among these animals, leopard, leopard cat, and Bengal tiger belong to family Felidae and golden jackal to family Canidae [1]. Leopard and Bengal tiger are in the red list of the International Union for Conservation of Nature and Natural resources as a vulnerable species [2]. Leopard cat and golden jackal are grouped as animals of least concern as their population is stable. Leopard is the most widespread wild cat species [1,3]. However, the population drastically reduced on account of poaching, habitat loss, and habitat fragmentation. Leopard’s skin color is tawny yellow and its fur presents densely packed dark irregular black rosettes [4]. Leopard cat is a small wild cat and its skin had yellowish ground color. Two distinct dark stripes extending between eyes and ears, black spots on dorsum are its characteristic features. Usually, the black spots are arranged in two to four rows on the dorsum of animal. Tigers are exclusively distributed in Asia, and Bengal tiger is also an endangered species. It is one of the largest extant wild cats. Its coat color is yellow to light orange and presents dark brown or black stripes. The tail is orange with black rings. Jackals are medium-sized omnivorous mammals, and golden jackal is the species distributed extensively in south Asia, including India [4].

Indiscriminate poaching and killing of wild mammals, for the production of hide, teeth, meat, and other items, is an undesirable truth in many parts of the world. The hide and trophies of wild animals will fetch exorbitant prices in the grey market. Such instances had resulted in the production and sale of fake products. The structure of the skin and its appendages such as hair is diverse among domestic and wild mammals. A baseline data on the skin of wild animals are essential to distinguish original and fake products. A morphometric study of hair of different breeds of dog had been recorded [5].

Histomorphological features of the skin of wild animals such as leopard and Bengal tiger are scarce, while the records on leopard cat and golden jackal are lacking. Hence, the present study was conducted to establish the histological features of the skin of leopard, leopard cat, Bengal tiger, and golden jackal.

## Materials and Methods

### Ethical approval

Permission for the conduct of the study was obtained from Chief Wildlife Warden, Kerala, as per order no. KFDHQ-915/2019-CWW/WL10 dated 11/03/2019.

### Study period

The study was conducted during the period from April 2019 to October 2019.

### Samples collection and processing

The skin samples of leopard (n=3), leopard cat (n=4), Bengal tiger (n=3), and golden jackal (n=4) were collected from the Department of Pathology, College of Veterinary and Animal Sciences, Pookode, when postmortem examination of the animals was carried out. Fresh skin samples of 2×2 cm size were taken from the lateral aspect of neck and abdomen; fixed in neutral buffered formalin for 48 h, and processed for routine paraffin embedding. Horizontal and vertical sections of 5 µm thickness were taken. The sections were stained by Hematoxylin and Eosin and Gomori’s one-step trichrome method [6]. Mounted the sections with DPX, and observed at 50, 150 and 500 magnifications under Olympus binocular research microscope.

## Results

**Figure-1:**
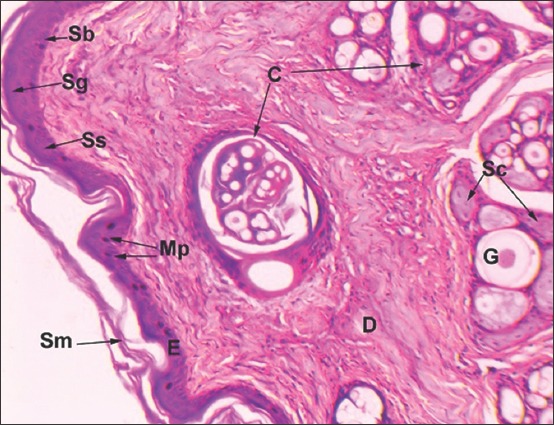
Vertical section of leopard skin: E-Epidermis, Sb-Stratum basale, Ss-Stratum spinosum, Sm-Stratum corneum D-Dermis, G-Primary Guard hair, C-Compound hair follicle, Sc-Sebaceous gland, Mp-Melanin pigment (Hematoxylin and Eosin ×150).

**Figure-2:**
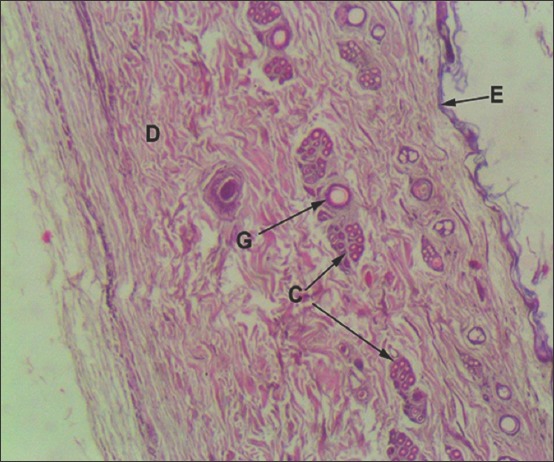
Vertical section of Leopard cat skin: E-Epithelium, D-Dermis, G-Primary Guard hair, C-Compound hair follicle (Hematoxylin and Eosin ×60).

**Figure-3:**
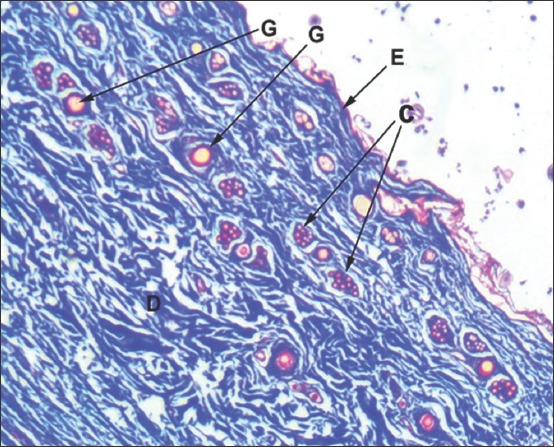
Vertical section of Leopard cat skin: E-Epithelium, D-Dermis, G-Primary Guard hair, C-Compound hair follicle (Masson’s trichrome ×60).

**Figure-4:**
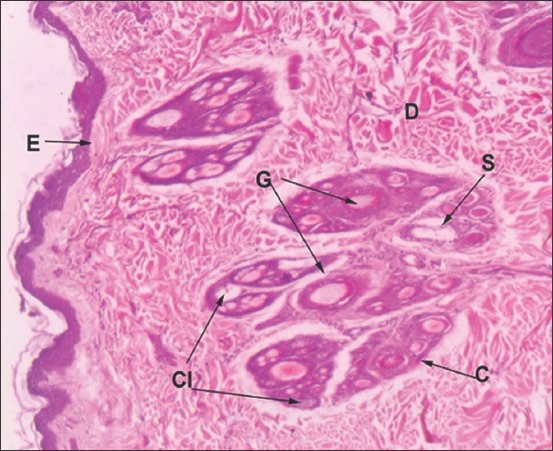
Vertical section of Bengal tiger skin: E-Epithelium, D-Dermis, Cl-Cluster of hair follicle, G-Primary Guard hair, C-Compound hair follicle, S-Sweat gland (Hematoxylin and Eosin ×60).

Skin comprised an outer epidermis and inner dermis in all the species studied. Epidermis was made up of keratinized stratified squamous epithelium (Figures-1-4). The epithelium presented five layers, namely, stratum basale, stratum spinosum, stratum granulosum, stratum lucidum, and stratum corneum. However, the occurrence of various strata as well as number of cell layers in each stratum varied in different species.

### Skin of leopard

All the typical layers of stratified squamous epithelium except stratum lucidum were present in the epidermis. A single layer of tall columnar cells enclosing oval nuclei constituted the stratum basale, whereas four to five rows of large polygonal cells, which enclose large round to oval nuclei created the stratum spinosum. The presence of melanocytes and abundant melanin pigments in the stratum basale was a salient feature. Stratum granulosum was two to three cell layers thick and enclosed basophilic keratohyalin granules. Five to six layers of flat squamous cells without nuclei and organelles constituted the keratinized stratum corneum (Figure-1).

**Figure-5:**
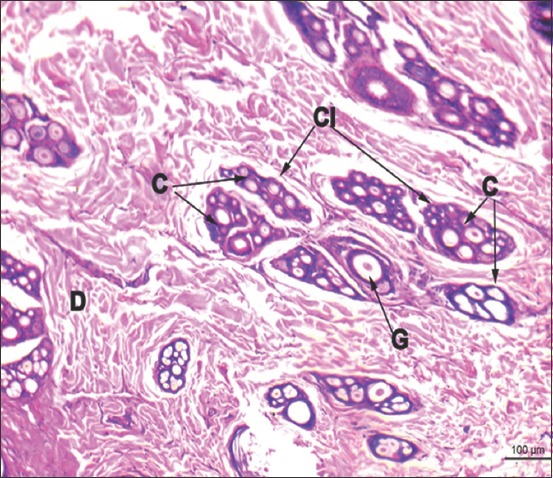
Horizontal section of leopard skin: D-Dermis, G-Primary Guard hair, Cl-Cluster of hair follicle, C-Compound hair follicle (Hematoxylin and Eosin ×60).

Dense irregular connective tissue composed of mainly collagen fibers and enclosing hair follicles, sweat, and sebaceous glands formed a thick dermis below the epidermis. Typically, the dermis was divisible into two zones: A thin, superficial papillary layer and a thick, and deep reticular layer. The papillary layer was formed by loose connective tissue and the reticular layer by dense irregular connective tissue. The dermis was not distinctly demarcated into papillary and reticular layers. Hair follicles were distributed uniformly in the dermis. The pattern of arrangement revealed a single large primary guard hair follicle delimited by five to seven compound hair follicles (Figure-5). The whole arrangement had an oval outline. Each compound follicle, in turn, comprised two to three coarse primary hairs and eight to 14 fine secondary hairs. Large branched alveolar sebaceous glands were present in association with primary follicles (Figure-5). The cells lining the periphery of the glands were squamous to cuboidal type. Lightly stained larger, polygonal cells enclosing centrally placed spherical nuclei occupied the interior of gland. In the center, nuclei appeared heterochromatic and pyknotic. Several acini of sebaceous glands were also present within the compound hair follicles in association with primary and secondary hair follicles. Arrectores pilorum muscle and simple tubular merocrine sweat glands were seen in association with the prominent large guard hair. The sweat glands were lined by simple cuboidal epithelium and were located in the superficial dermis.

### Skin of leopard cat

Epidermis of leopard cat skin illustrated only three layers, namely, stratum basale, stratum spinosum, and stratum corneum (Figures-2 and 3). Stratum granulosum and stratum lucidum were absent. Only a single row of nucleated cells was present in the stratum basale and stratum spinosum. The cells were cuboidal in the stratum basale while they were round to oval in the spinosum layer. The thin keratinized stratum corneum was made up of flat squamous cells.

Dense irregular connective tissue constituted the dermis. A very thin less densely packed superficial zone created papillary layer below the epidermis. Hair follicles presented uniform distribution. Dermis enclosed primary guard hair and compound hair follicles (Figures-2 and 3). The hair follicle pattern revealed a central primary guard hair encircled by two to five compound hair follicles. Each compound follicle, in turn, comprised one or two coarse primary hairs and six to ten fine secondary hairs (Figures-2 and 3). Small alveolar branched sebaceous glands were observed in association with the primary guard hair. Two or three sebaceous gland acini were present within a compound follicle. Arrectores pilorum muscle and a few simple tubular sweat glands lined by simple columnar cells were seen in association with the large guard hair.

### Skin of Bengal tiger

In the epidermis, all layers of the stratified squamous epithelium, except stratum lucidum, were present (Figure-4). Cells of stratum basale were low columnar. Stratum spinosum was formed by three to four rows of round cells. Stratum granulosum layer was comprised of two to three nucleated cell layers and enclosed basophilic keratohyalin granules. Keratinized stratum corneum was comparatively thicker and was made up of five to six layers of flat squamous cells.

**Figure-6:**
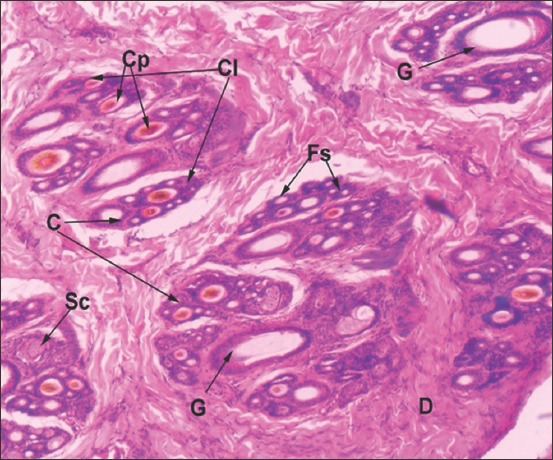
Horizontal section of Bengal tiger skin: D-Dermis, Cl-Cluster of hair follicle, G-Primary Guard hair, C-Compound hair follicle, Cp-Coarse primary hair follicles; Fs-Fine secondary hair follicles, Sc-Sebaceous gland (Hematoxylin and Eosin ×60).

Dermis contained compound follicles (Figures-4 and 6). Dermis contained one large primary guard hair surrounded by three to seven compound follicles. Each compound follicle contained one to two coarse primary hair follicles; six to twenty fine secondary hair follicles and large saccular sebaceous glands (Figures-4 and 6). Fine elastic and collagen tissue fibers surrounded individual hair follicles within a compound follicle. Each compound follicle was well-encapsulated by dense irregular connective tissue. Sebaceous glands were observed in association with guard primary hair follicles and coarse primary hair follicles. Saccular apocrine type sweat glands were located deep in the dermis in association with primary hair follicle (Figure-4). The glands were lined by simple columnar cells and presented apical blebs. Arrectores pilorum muscles were present near the follicles.

### Skin of golden jackal

In golden jackal, only four layers, namely, stratum basale, stratum spinosum, stratum granulosum, and stratum corneum were present in the epidermis and the stratum lucidum was lacking. A single layer of cuboidal cells constituted the stratum basale. Two to three rows of oval cells were observed in the spinosum layer. One to two rows of almost flat cells made up the stratum granulosum. Stratum corneum was a thin layer made by dead flat cells.

**Figure-7:**
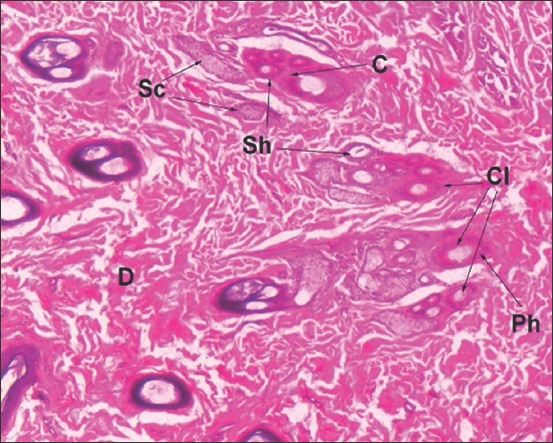
Horizontal section of golden jackal skin: D-Dermis, Cl-Cluster of hair follicle, C-Compound hair follicle, Ph-Primary hair follicle, Sh-Secondary hair follicles, Sc-Sebaceous gland (Hematoxylin and Eosin ×60).

**Figure-8:**
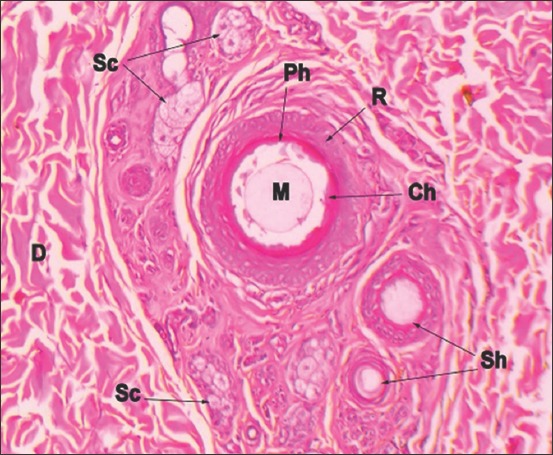
Horizontal section of golden jackal skin showing a compound hair follicle D-Dermis, Ph-Primary hair follicle, Sh-Secondary hair follicles, Sc-Sebaceous gland, M-Medulla of hair follicle, Ch-Cortex of hair follicle, R-Root sheath of hair follicle (Hematoxylin and Eosin ×150).

Dense irregular connective tissue was observed in the dermis. It was less densely packed below the epidermis than in the deeper zone. Compound follicles were observed in the dermis (Figures-7 and 8). However, the pattern and number of follicles were entirely different from other felines of the study. The compound follicles were arranged as clusters of three, wherein central follicle was larger than the lateral follicles (Figure-7). Each compound follicle comprised of single long primary hair and six to eight smaller secondary hairs. Compound follicles were encapsulated by connective tissue. Primary and secondary hair follicles were associated with large compound saccular sebaceous glands (Figure-8). Apocrine sweat glands lined by columnar cells and possessing apical blebs were observed in association with the primary hair follicle.

## Discussion

Keratinized stratified squamous epithelium made up epidermis in domestic dog, domestic cat, Sphynx cat, leopard, and Bengal tiger [7-10]. All the typical layers were reported in Bengal tiger skin [10]. However, epidermal layers except stratum lucidum were stated in leopard, the domestic cat, and Sphynx cat [8,10], and the absence of stratum granulosum and stratum lucidum reported in the domestic dog [10] and the domestic cat [11]. In the study, stratum lucidum was not discernible in leopard, Bengal tiger, and golden jackal, whereas stratum granulosum and stratum lucidum were not observed in leopard cat. One to two cell layers constituted stratum corneum in leopard [10]. However, five to six layers of squamous cells were observed in the study. Skin form different regions of body vary considerably in thickness and may account for variation in the number of layers and number of cells within each layer observed in the study. Melanin pigments, which were previously reported in domestic cat [10] were not discernible in leopard cat.

Demarcation of dermis into a papillary layer and a reticular layer was reported in leopard and in domestic dog [10] and a very thin papillary layer in domestic cat [12]. However, in the present study, a clear distinction was present in golden jackal and demarcation was less distinct in leopard cat and leopard.

Compound follicles are characteristic features of domestic dog and domestic cat [11,12], leopard, Bengal tiger [7,10], and Sphynx cat [8]. However, the pattern and number of follicles varied greatly in the species included in the study. One leading primary hair, fine guard hairs, and fine secondary hairs were described in domestic cat [11]. Single large guard hair surrounded by two to five compound follicles was present in domestic cat [9]. Less number of leading primary hairs reported in domestic cat [11].

The study revealed existence of guard primary hair follicles in leopard, leopard cat, and Bengal tiger, which had been described in the domestic cat and in Sphynx cat [8,9]. Guard and under fur hair types were recorded in leopard [3]. Even though compound hair follicles were described in leopard [10], number of compound follicles within a cluster, existence of coarse primary hair follicles, and the number of different types of hairs within a compound follicle were not mentioned. Furthermore, only a smaller number of hair follicles were described within a compound follicle [7,10]. In the case of Bengal tiger, even presence of guard primary hair follicles and coarse primary hair follicles was not mentioned [7,10]. Hair follicles arrangement in leopard, leopard cat, and Bengal tiger revealed in the study, in general, correspond to domestic cat [9]. However, great variation existed in the number of coarse and fine secondary hair follicles among leopard, leopard cat, and Bengal tiger. Further, the data obtained in wild cats obviously deviated from the records in the domestic cat [9]. Compound follicles occurred in clusters of three, and the central one was larger than the laterals in dog [9,12]. The compound follicles of golden jackal were similar to the domestic dog and were well-encapsulated by connective tissue as described in dog [9]. No records on hair follicle pattern in leopard cat and golden jackal were available. The pattern of hair follicle arrangement and number of various types of hair follicle were found to be entirely different in all the species of the present study.

Large sebaceous glands were observed in leopard, Bengal tiger, and golden jackal, which may have a function in the lubrication of hair and skin [12]. The small sebaceous glands observed in leopard cat are comparable to the glands reported in the domestic cat [12]. Atrophic, rudimentary sweat glands were present in the domestic dog and the domestic cat [11]. The glands in dogs and cats were described as tortuous and apocrine type [9]. The occurrence of apical blebs on the lining cells of sweat glands indicates apocrine type activity in Bengal tiger and golden jackal. The apocrine sweat glands may function in the production of fat and pheromones [11].

## Conclusion

Histological structure of skin in leopard, leopard cat, Bengal tiger, and golden jackal varied greatly. Variation existed in the number and type of layers in the epidermis as well as pattern of arrangement of hair follicles. All species were characterized by compound hair follicles. The study provided baseline data on the skin histology and hair follicle pattern, and it may be useful in the identification of unknown skin samples of wild carnivores. Further, the data form a valuable basis for comparative histology of wild carnivores.

## Authors’ Contributions

CVR, HSP, and MP designed the study. CVR, KS, and PMD collected samples. CVR, KS, and PS did the laboratory works. CVR, HSP, and PMD drafted the manuscript. All the authors participated in the drafting, revision of the manuscript, and approved the final version.
